# High immunity and low mortality after Omicron and mass event in Cameroon despite low vaccination

**DOI:** 10.4102/jphia.v15i1.649

**Published:** 2024-11-07

**Authors:** Yap Boum, Lucrece Matchim, Dominique K. Guimsop, Bongkiyung D. Buri, Lisa M. Bebell, Yuya S.F. Jaudel, Fai K.G. Njuwa, Daniel B. Danirla, Eric Youm, Rodrigue Ntone, Claudric Roosevelt Tchame, Dora Tchiasso, Rachelle Essaka, Justin B. Eyong, Audrey Ngosso, Herwin Nanda, Nsaibirni R. Fondze, Mark Ndifon Ndifon, Lucrèce Eteki, Yonta F.C. Ghislain, Bruno Yannick Eyenga Messi, Hamadou Moustapha, Moustafa Hamdja, René Ghislain Essomba, Nadia Mandeng, Tamakloe A.K. Modeste, Anne-Cécile Zoung-Kani Bisseck, Sara Irène Eyangoh, Richard Njouom, Marie Claire Okomo, Linda Esso, Epee Emilienne, Georges-Alain Etoundi Mballa

**Affiliations:** 1 Public Health Emergency Operation Center, Ministry of Public Health, Yaoundé, Cameroon; 2 Epicentre, Yaoundé, Cameroon; 3 Faculty of Biomedical Medicine and Science, University of Yaoundé I, Yaoundé, Cameroon; 4 Department for the Control of Disease, Epidemics and Pandemics, Ministry of Public Health, Yaoundé, Cameroon; 5 Western Africa Regional Coordination Center, Africa Centers for Disease Control, Abuja, Nigeria; 6 Department of Medicine, Massachusetts General Hospital, and Harvard Medical School, Boston, Massachusetts, United States of America; 7 Epicentre, Paris, France; 8 Laboratoire du Lac, Yaoundé, Cameroon; 9 National Public Health Laboratory, Yaoundé, Cameroon; 10 Médecins Sans Frontières Suisse, Yaoundé, Cameroon; 11 Health Operations Research Division, Ministry of Public Health, Yaoundé, Cameroon; 12 Centre Pasteur du Cameroon, Yaoundé, Cameroon; 13 Department for the Control of Disease, Epidemics and Pandemics, Yaoundé, Cameroon

**Keywords:** seroprevalence, SARS-CoV-2, mortality, immunity, Africa

## Abstract

**Background:**

Little is known about the evolution of severe acute respiratory syndrome coronavirus 2 (SARS-CoV-2) immunity in African communities.

**Aim:**

We evaluated changes in anti-SARS-CoV-2 antibodies, mortality and vaccination status in Cameroon between August 2021 and September 2022 to begin describing the evolution of the pandemic in Africa.

**Setting:**

The study was conducted across Cameroon’s 10 regional capitals, between 2021 and 2022 as the country hosted a mass gathering.

**Methods:**

We conducted a cross-sectional population-based survey in 2022, including SARS-CoV-2 seroprevalence testing and retrospective mortality estimation using two-stage cluster sampling. We estimated and compared seroprevalence and crude mortality rates (CMR) to a survey conducted in 2021 using the same methodology.

**Results:**

We performed serologic testing on 8400 individuals and collected mortality data from 22 314 individuals. Approximately 5% in each survey reported SARS-CoV-2-vaccination. Rapid diagnostic test-based seroprevalence increased from 11.2% (95% confidence interval [CI]: 10–12.5) to 59.8% (95% CI: 58.3–61.2) between 2021 and 2022, despite no increase in the proportion vaccinated. The CMR decreased from 0.17 to 0.06 deaths per 10 000 persons per day between 2021 and 2022. In 2022, no deaths were reportedly attributable to COVID-19 as compared to 17 deaths in 2021.

**Conclusion:**

Over a 12-month period encompassing two waves of omicron variant SARS-CoV-2 and a mass gathering, SARS-CoV-2 seropositivity in Cameroon approached 60%, and deaths declined despite low vaccination coverage.

**Contribution:**

This study challenges the assumption that high immunisation coverage is the sole determinant of epidemic control in the African context and encourages policymakers to increasingly rely on local research when designing response strategies for more effective outbreak management.

## Introduction

At the onset of the severe acute respiratory syndrome coronavirus 2 (SARS-CoV-2) pandemic, several predictions forecasted that Africa could face the worst consequences of the epidemic.^1,2^ The continent was perceived as particularly vulnerable because of weak health systems, fragile infrastructure, a shortage of trained personnel, poor access to healthcare delivery, low hygiene standards and a rising prevalence of comorbidities. However, as of 14 July 2023, Africa counted only 175 399 deaths, registering about 3% of global COVID-19 mortality, despite being home to 16.7% of the world’s population.^3,4^ Although there have been significant challenges related to under-testing and underreporting across the continent,^5^ these figures are still far short of the predicted scenario.

**FIGURE 1:**
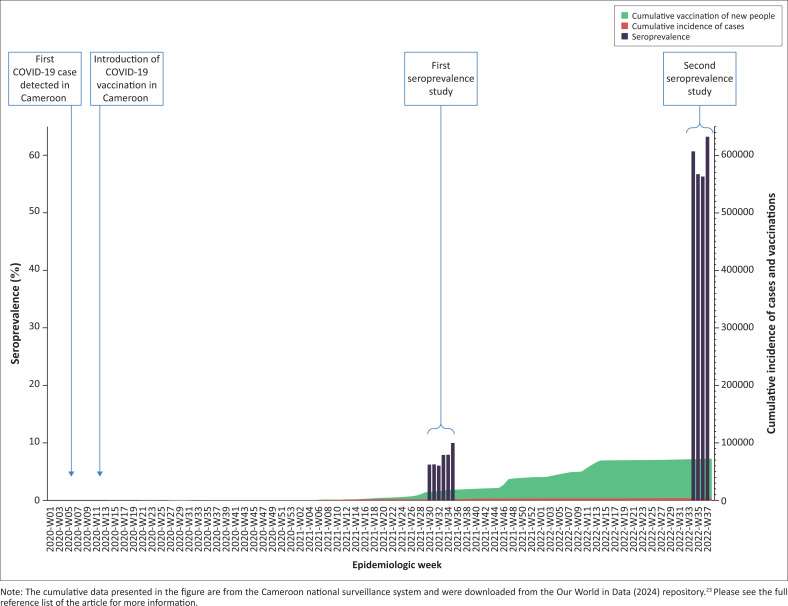
Unadjusted severe acute respiratory syndrome coronavirus 2 antibody seroprevalence during the two surveys and cumulative incidence of confirmed cases and vaccination in Cameroon, March 2020 – September 2022.

In Cameroon, pandemic control focused on surveillance, Public Health and Social Measures (PHSM), strengthening case detection and management capacities and later, large-scale vaccination.^6^ National surveillance data reported a cumulative number of only 123 993 confirmed cases of COVID-19 with 1965 deaths by 14 September 2022 (Figure 1), with an epidemiologic curve revealing five separate attack waves since March 2020.^7^ The later waves in December 2021 and August 2022 flanked a major football event held in January 2022 in Cameroon, the African Cup of Nations, which attracted millions of visitors. The December 2021 wave was dominated by the B.1.1.529 omicron variant, and the August 2022 wave by the BA.4 and BA.5 omicron variants, remarkable for their increased transmissibility and virulence compared to prior omicron variants.^8^ During this period, COVID-19 mortality in Cameroon remained low despite an overall vaccination rate of < 10%.^7^

To better understand the low COVID-19 mortality over time in the face of low vaccination rates, we aimed to evaluate population-level immunity in Cameroon and its dynamics over time, including the potential influence of large gatherings such as the African Cup of Nations, which took place in Cameroon 6 months prior to the study. We also hypothesised that a large-scale sero-epidemiologic study would overcome the limitations of currently-available surveillance data, including underreporting and lack of countrywide representation.^9,10^

To address the apparent paradox in SARS-CoV-2 vaccination and mortality rates in sub-Saharan Africa, we carried out a population-based serosurvey and retrospective mortality estimation across the 10 regions of Cameroon, following the first survey conducted in 2021^11^ to describe the progression of the SARS-CoV-2 population immunity. We aimed to fill gaps in knowledge about pandemic evolution over time in Africa in order to inform future public health objectives and adjust mitigating strategies for future pandemics.

## Research methods and design

### Study design and population

Our study consisted of cross-sectional population-based surveys in the 10 regional capitals in Cameroon, a Central African country with an area of 475 440 km^2^ and a population of approximately 27 million inhabitants with a moderate population density of 57 people per km^2^.^12^ This second survey was conducted from 25 August 2022 to 15 September 2022 using the same protocol as the first survey implemented from 27 July 2021 to 31 August 2021.^11^ As the previous survey implemented in 2021,^11^ this survey had two components: (1) measurement of SARS-CoV-2 seroprevalence, and (2) retrospective mortality estimation.

### Sample size

The sample size calculation follows the methodology described in the 2021 seroprevalence survey.^11^ For the seroprevalence measurement, assuming 20% seroprevalence in the high-transmission stratum and 10% in the low-transmission stratum, precision of ± 5% for each age group (< 20 years, 20–39 years and 40+ years), type 1 error of 5%, and 5% inconclusive test results; we calculated a sample size for serologic testing of 1033 individuals per age group in the high-transmission stratum and 291 individuals per age group in the low-transmission stratum. This yielded an overall sample size of 6000 participants in the high-transmission stratum and 1691 participants in the low-transmission stratum. Considering an average household size of five people, this led to a total sample size of 1200 households in the high-transmission stratum and 339 households in the low-transmission stratum. With a cluster comprising 30 households, we included first 10 households (for high-transmission) and five households (for low-transmission) within each cluster, for the serological evaluation, providing a ~5% margin for individuals who declined or were unable to participate.

To determine the sample size for the mortality estimation, we first of all estimated expected changes in the crude mortality rate (CMR) because of COVID-19 using simulations. We assumed age group specific CMRs for Cameroon using 2016 World Health Organization (WHO) data, and estimated COVID-19 infection fatality rates (IFR) by age group as described by O’Driscoll and colleagues.^13^ The assumptions outlined in this study were derived from the established protocol and assumptions of the initial study, which were previously published in *Scientific African*.^11^ The aim of this study was to compare the main findings between these two periods. For further details on the specific assumptions made to estimate the sample size, we refer to the Methods section of the first study.^11^ The required sample size derived from the assumptions was 28 077 participants (5616 households).

### Household selection

We used a two-stage cluster sampling method based on random geo-points to select households.^14^ In the first stage, we used the Epicentre Geo sampler tool^14^ to select 198 clusters (neighbourhoods), with selection probability proportional to population size of the area in which the cluster was located, carefully excluding non-residential areas. In the mortality survey, we aimed to include 30 households per cluster. For the seroprevalence survey, we used the national COVID-19 surveillance data to divide the population of Cameroon into two strata: a high-transmission stratum corresponding to an incidence ≥ 0.5 cases per 1000 individuals (a case was defined based on a positive rapid diagnostic test [RDT] or polymerase chain reaction [PCR] result), including the Centre, Littoral, Northwest, West, East and South regions; and a low-transmission stratum corresponding to < 0.5 cases per 1000 individuals, including the Adamawa, Southwest, North and Far North regions. We then set to include the first 10 and first 5 households per cluster for the high- and low-transmission strata, respectively, into the seroprevalence survey.

To select individual households for the serosurvey, we used two methods, depending on their appropriateness for a given cluster. We used the first method (1) for most clusters. However, for 37 clusters located in rural areas or conflict zones, where most of the preselected households were hard-to-reach, we used the second method (2):

1. We randomly preselected 30 households (with 10 alternate households in case of inaccessibility or declined study participation) in each cluster using the Epicentre Geo sampler tool. We retrieved the Global Positioning System (GPS) coordinates of each household and uploaded them to tablets through the Open Street Map Automated Navigation Directions (OsmAnd) application.^15^ Our teams then used the application to identify the households. Specifically, within each cluster, our objective was to visit a total of 30 households. To achieve this, we preloaded the GPS coordinates for 40 households per cluster at once and numbered them sequentially from the ‘cluster center’ (a randomly preselected point generated on the cluster map) outwards. During the fieldwork, the households were visited consecutively, starting from the household number 1. If any of the initially visited households were inaccessible, the survey team would proceed to the next available numbered household within our preloaded list to replace the inaccessible one until the required number of 30 responding households was achieved.
2. We selected the first household closest to the cluster center. After including this first household, the survey team selected the next household using a systematic procedure: while standing at the front door of the first household and looking outward, they moved left and skipped four households (while remaining within neighbourhood boundaries) and selected the fifth household for the next interview. We systematically repeated this method until we included the required number of households. If a selected household declined to participate or was empty, the team documented the event, and continued with the same methodology to identify the next household until they reached the required size.

When more than one household was in the same compound, house, or building, the households were numbered, and a single one of them was randomly included. We included all people residing in the selected households. We excluded people who declined to participate in the study and those aged less than 21 years with no adult representative.

### Procedures

For seroprevalence measurement, we collected data using a structured electronic questionnaire and collected fingerstick blood samples to perform SARS-CoV-2 antibody testing in the participants’ households using antibody RDTs. We collected dried blood spots (DBS) from participants for further analysis in the laboratory using enzyme-linked fluorescent assays (ELFA).

For mortality estimation, we interviewed an adult household representative using the WHO guidelines for verbal autopsy to identify COVID-19 and non-COVID-19 deaths.^16^ The questionnaire included housing characteristics, demographics, COVID-19 vaccination status and information on deaths that occurred during a specified recall period (01 September 2021 – 31 August 2022).

### Laboratory analysis

We used four different rapid diagnostic testing kits to detect immunoglobulin G (IgG) and immunoglobulin M (IgM) anti-SARS-CoV-2 antibodies, including: (1) Wondfo^®^ lateral-flow immunochromatographic (IC) IgM/IgG antibodies test (Guangzhou Wondfo Biotech Co., Ltd, China),^17,18^ (2) Encode COVID-19 IgG/IgM Rapid test device (Zhuhai Encode Medical Engineering Co., Zhuhai, China),^10^ (3) Hightop COVID-19 IgG/IgM Rapid test device (Qingdao Hightop Biotech Co., Ltd, China)^17^ and (4) Rightsign™ COVID-19 IgG/IgM Rapid Test Cassette (Hangzhou Biotest Biotech Co., Ltd. No. 17, Futai Road, Zhongtai Street, Yuhang District, Hangzhou, China).^19^ We used only one of the four RDT kits to test each individual, using the brand available at the time of testing. Comparative performances of used RDT assays are provided in Table 1.

**TABLE 1:** Comparative performance of serological test assays performed in accordance with the manufacturer’s protocol with reverse transcription polymerase chain reaction, at all sampling time points post symptom onset.

Test assay	Performance characteristic								Total number	
	Sensitivity		Specificity		Positive predictive value		Negative predictive value			
	%	95% CI	%	95% CI	%	95% CI	%	95% CI	Samples	Patients
Wondfo†^18^	45.2	-	81.8	-	80.5	-	47.4	-	-	-
Wondfo†^17^	68.6	60.1–76.3	97.8	92.4–99.7	97.9	92.7–99.8	67.7	59.0–75.5	229	183
Hightop IgM^17^	39.0	30.7–47.7	100.0	96.1–100	100.0	93.3–100	52.6	44.9–60.2	228	182
Hightop IgG^17^	58.8	50.7–67.2	100.0	96.1–100	100.0	95.6–100	62.2	53.8–70.0	228	182
Hightop: IgM or IgG^17^	61.0	52.3–69.3	100.0	96.1–100	100.0	95.7–100	63.4	55.1,71.3	228	182
Encode IgM and IgG^10^	93.4	87.8–96.9	99.0	94.6–100.0	-	-	-	-	-	-
**RightSign: IgM and IgG^19^**	100.0	88.7–100	100.0	95.4–100	-	-	-	-	-	-
Sensitivity	-	-	-	-	-	-	-	-	30	30
Specificity	-	-	-	-	-	-	-	-	80	80

*Source:* Adapted from Eyong J, Fai KN, Nikolay B, et al. Nationwide retrospective mortality and seroprevalence of SARS-CoV-2 antibodies in Cameroon. Sci Afr. 2023;22:e01925. https://doi.org/10.1016/j.sciaf.2023.e01925

Note: Please see the full reference list of the article, for more information.

IgM, immunoglobulin M; IgG, immunoglobulin G; CI, confidence interval.

† , Single test line captures IgM and IgG antibodies.

We made DBS from the fingerstick blood samples and transported them to Yaoundé Teaching Hospital for ELFA testing. We used the Biomerieux ELFA kit^20^ to measure anti-SARS-CoV-2 immune globulin (Ig) G2 antibodies from DBS. We performed all testing according to each manufacturer’s instructions. We used a cut-off value of ≥ 1 to define seropositivity with serum ELFA testing, according to the manufacturer’s instructions. As we used DBS, which have a lower antibody concentration than serum^9^ as used in the 2021 survey, we estimated a cut-off value of ≥ 0.14 by collecting sera and whole blood samples from six individuals and preparing serial dilutions from both sample types: We used diluted whole blood to prepare DBS and analysed diluted sera and diluted DBS for SARS-CoV-2 antibodies (IgG-2). We used these values to compute the DBS cut-off values corresponding to the cut-off value of ≥ 1 for undiluted serum.

We defined a positive SARS-CoV-2 RDT result for seroprevalence estimation as either, or both, positive IgM or positive IgG. We considered an ELFA DBS assay positive when the relative fluorescent value was ≥ 0.14. We used positive RDT and ELFA results to estimate the overall and regional seroprevalence of SARS-CoV-2 antibodies.

### Statistical analysis

We accounted for the sampling design of the survey using the ‘survey’ package (svydesign and svyciprop functions) of *R* version 4.2. Seroprevalence and 95% CIs were estimated using the survey package in *R*, weighting for demographic differences between the surveys samples and the general population and adjusting for the design effect.

We used the recent households deaths (RHD) method to estimate general mortality. As directed by guidance on mortality estimates from retrospective households surveys,^21,22^ we calculated mortality rates (expressed as deaths/10 000 people per day) with 95% CI as the number of deaths over the total person-time of all individuals times 10 000. We calculated person-time for each household member using the period between the beginning of the recall period, date of arrival in the household (for those that later joined the household), or date of birth (for those born after the start of the recall period) and the end of the recall period. Cause-specific fractions were then determined as the proportion of all deaths that were attributable to a specific cause of death (CoD) as per respondents’ reports.

### Ethical considerations

The study was approved by the National Ethics Committee for Human Health Research in Cameroon (number 2021/07/1371/CE/CNERSH/SP) and received administrative authorisation from the Ministry of Public Health of Cameroon. We conducted all study procedures according to the Helsinki Declaration and followed Good Clinical Practice guidelines. We obtained written informed consent from all adult participants and from the parents or a legal representative of all people younger than 21 years. Written assent was obtained from all participants aged < 21 years. To protect participant privacy, data were anonymised, personal identifiers like names were removed or replaced with codes. Access to identifiable data was restricted to essential personnel only. Participants’ health and safety was priority. Participants with positive test results were advised to visit recommended health facilities for polymerase chain reaction (PCR) confirmation or to seek appropriate medical care. Additionally, they were briefed on Public Health and Social Measures (PHSM) and informed about critical warning signs that would necessitate immediate medical attention.

## Results

A total of 8400 individuals from 3325 households took part in the 2022 seroprevalence measurement based on RDT, compared to the 5730 retrieved from the 2021 study.

Out of the 8400 samples, 5094 (60.6%) tested positive for either antibody type (IgM or IgG), compared to 757 (13.2%) in 2021; 3889 (46.3%) tested positive for IgG only compared to 346 (6.0%) in 2021; while 436 (5.2%) tested positive for IgM only, compared to 60 (1%) in 2021. Rapid Diagnostic Test and ELFA were extensively compared in the in the first phase study and found to be consistent. In the remaining of this study, only adjusted RDT-based seroprevalence estimates will be presented.

While the cumulative incidence of cases showed a very slow increase from 2970 cases per million in July 2021 to 4428 cases per million in September 2022, and the cumulative vaccination of new people only tripled to 67 938 cases per million (Figure 1), the overall seroprevalence of SARS-CoV-2 antibodies increased nearly sixfold from 11.2% (95% CI: 10.0–12.5) to 59.8% (95% CI: 58.3–61.2) during the same period (Table 2).

**TABLE 2:** Unadjusted seroprevalence of anti-severe acute respiratory syndrome coronavirus 2 antibodies in Cameroon across the two surveys: Rapid diagnostic test and enzyme-linked fluorescence assays based results.

Test result	Survey 1:^11^ 27 July 2021 – 31 August 2021				Survey 2: 25 August 2022 – 15 September 2022			
	Samples		Seroprevalence		Samples		Seroprevalence	
	*n*	*N*	%	95% CI	*n*	*N*	%	95% CI
**RDT**								
IgG or IgM	757	5730	13.2	12.3–14.1	5094	8400	60.6	59.6–61.7
IgG only	346	5730	6.0	5.4–6.7	3889	8400	46.3	45.2–47.4
IgM only	60	5730	1.0	0.8–1.3	436	8400	5.2	4.7–5.7
Both IgG and IgM	351	5730	6.1	5.5–6.8	769	8400	9.2	8.5–9.8
Invalid	21	5730	0.4	0.2–0.6	15	8400	0.2	0.1–0.4
**ELFA**								
IgG	205	5844	16.7	15.8–17.7	2951	4107	71.9	70.4–73.2

Note: Please see the full reference list of the article, for more information. Sizes and frequencies in this table are unweighted.

*n*, number of positive; *N*, total; RDT, rapid diagnostic test; ELFA, enzyme-linked fluorescent assay; IgM, immunoglobulin M; IgG, immunoglobulin G; CI, confidence interval.^20^

Table 3 describes seroprevalence samples population and presents RDT-based adjusted seroprevalence by across both surveys. Overall, females comprised 55% (*n* = 4621) of the 2022 sample whose median age was 27 (interquartile range [IQR]: 17–39) years. Participants aged < 20 years comprised 28.0% (vs. 18.5% in the 2021 seroprevalence measurement survey) of the sample, 20–34 years comprised 39.0% (2021: 41.9%), 35–49 years 18.4% (2021: 21.3%) and ≥ 50 years comprised 14.5% (2021: 18.3%). The regional distribution of the 2022 sample was also similar to the 2021 seroprevalence measurement survey with the Centre (30.9%, *n* = 2597), Littoral (31.8%, *n* = 2674) and Far North (6.9%, *n* = 582) being the most represented regions. The vaccination coverage from the 2022 sample was 5.1% (*n* = 390) compared to 5.9% from the 2021 sample.

**TABLE 3:** Adjusted seroprevalence of anti-severe acute respiratory syndrome coronavirus 2 antibodies by sociodemographic characteristics and vaccination status in Cameroon across the two surveys.a†

Characteristic	Survey 1:^11^ 27 July 2021 – 31 August 2021						Survey 2: 25 August 2022 – 15 September 2022					
	Samples population		Samples positivity		Seroprevalence		Samples population		Samples positivity		Seroprevalence	
	*N*	%	*n*	*N*	%	95% CI	*N*	%	*n*	*N*	%	95% CI
**Sex**												
Male	2594	45.3	368	2594	12.4	10.6–14.4	3775	45	2191	3775	57	55.1–58.9
Female	3136	54.7	389	3136	10.3	8.8–11.9	4621	55	2903	4621	62.1	60.4–63.9
**Age (years)**												
0–19	1058	18.5	88	1058	8.1	6–10.6	2345	28	1338	2345	56.8	54.3–59.3
20–34	2403	41.9	283	2403	10.6	9–12.4	3272	39.1	1961	3272	59.9	57.9–61.8
35–49	1220	21.3	189	1220	14.1	11.7–16.8	1542	18.4	979	1542	63.4	60.8–66
≥ 50	1049	18.3	197	1049	17.2	14.4–20.3	1216	14.5	807	1216	66.1	63.2–68.9
**Region**												
Adamawa	363	6.3	21	363	4.8	2.3–8.6	476	5.7	288	476	58.6	53–64.1
Center	1655	28.9	275	1655	16.4	14–18.9	2597	30.9	1530	2597	58	55.3–60.6
East	281	4.9	50	281	19.6	11.5–29.8	323	3.8	249	323	74.9	67.6–81.4
Far North	604	10.5	50	604	7.5	4.1–12.3	582	6.9	399	582	69.6	65.2–73.7
Littoral	1314	22.9	119	1314	8.3	6.6–10.3	2674	31.8	1506	2674	55.6	53–58.1
North	536	9.4	93	536	13.2	9.3–17.7	510	6.1	319	510	62.9	57.9–67.8
Northwest	341	6	39	341	9.8	6.4–14	416	5	309	416	73	67.8–77.8
West	306	5.3	54	306	17.2	12.7–22.5	471	5.6	278	471	57.1	50.7–63.4
South	151	2.6	27	151	13.2	8.4–19.2	166	2	97	166	56.7	47.2–65.9
Southwest	179	3.1	29	179	15.6	7.8–26.3	185	2.2	119	185	63.8	52.4–74.3
**Vaccination status**												
Vaccinated	312	5.9	138	312	44.9	22.3–69.1	390	5.1	287	390	67.6	59.7–74.8
Unvaccinated	5305	94.1	592	5305	9.5	8.4–10.7	7578	94.9	4527	7578	59.1	57.6–60.6

**Total**	**5730**	**100**	**757**	**5730**	**11.2**	**10–12.5**	**8400**	**100**	**5094**	**8400**	**59.8**	**58.3–61.2**

Note: Please see the full reference list of the article for more information.

*n*, Number of positive; *N*, total; CI, confidence interval.

† , Rapid diagnostic test-based results.

The seroprevalence observed from Survey 2 was statistically higher among females (62.1%, 95% CI: 60.4–63.9) than males (57.0%, 95% CI: 55.1–58.9), which was not the case during Survey 1. However, during both surveys, seroprevalence showed a progressive increase with age and was highest among the elderly (≥ 50 years). The East region had the highest seroprevalence in both surveys (74.9%, 95% CI: 67.6–81.4 in 2022 vs. 19.6%, 95% CI: 11.5–29.8 in 2021). The lowest seroprevalence during Survey 2 was observed in the Littoral (55.6%, 95% CI: 53–58.1) and the South (56.7%, 95% CI: 47.2–65.9).

There was no statistical difference between the seroprevalence among vaccinated (67.6%, 95% CI: 59.7–74.8) and unvaccinated people (59.1%, 95% CI: 57.6–60.6) during Survey 2, while during Survey 1, the seroprevalence in vaccinated people (44.9%, 95% CI: 22.3–69.1) was about five times higher than that in unvaccinated people (9.5%, 95% CI: 8.4–10.7)

**FIGURE 2:**
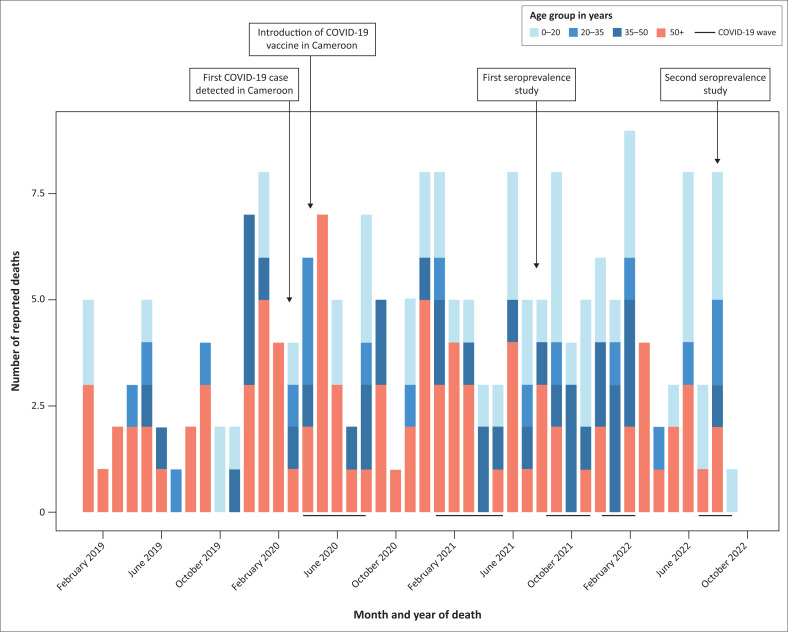
Monthly number of reported deaths by age group in Cameroon, from January 2019 to November 2022.

A total of 27 105 individuals from 5689 households took part in the mortality estimation survey. The median age was 23 (IQR: 12–35) years (30 [IQR: 22–43] years in 2021) and females comprised 52.9% of the sample (53.3% in 2021). Participants aged < 20 years comprised 41.6% of the sample (39.3% in 2021), 20–34 years 32.9% (32.0% in 2021), 35–49 years 14.6% (16.4% in 2021) and ≥ 50 years, 10.9% (12.3% in 2021). Overall, death occurrence was more frequent in the above 50 age group (Figure 2).

The CMR decreased from 0.17 (95% CI: 0.14–0.20) in 2021 to 0.06 (95% CI: 0.04–0.08) per 10 000 individuals in 2022, indicating a significant decrease in overall mortality (Table 4). In 2022, mortality did not significantly vary across both sexes (0.07, 95% CI: 0.04–0.1 vs. 0.05, 95% CI: 0.02–0.07). The mortality rate in 2022 was lowest in the 20–35 years age group (0.03, 95% CI: 0–0.06) and progressively increased across higher age groups. A similar pattern was observed in 2021. However, while in 2021 the mortality was significantly higher in the elderly (0.76, 95% CI: 0.61–0.95) than in the 35–50 years age group (0.14, 95% CI: 0.08–0.21), this difference was not significant in 2022 (0.08, 95% CI: 0.02–0.15 vs 0.16, 95% CI: 0.06–0.26).

**TABLE 4:** Death counts and crude mortality rates by participant age and sex.

Characteristic	2021 deaths	2021^11^ CMR		2022 deaths	2022 CMR	
	*n*	Proportion (per 10 000)	95% CI	*n*	Proportion (per 10 000)	95% CI
Overall	177	0.17	0.14–0.20	65	0.06	0.04–0.08
**Sex**						
Male	97	0.21	0.16–0.26	37	0.07	0.04–0.1
Female	80	0.15	0.11–0.18	28	0.05	0.02–0.07
**Age (years)**						
0–20	29	0.08	0.04–0.11	24	0.05	0.02–0.08
20–35	20	0.06	0.03–0.1	7	0.03	0.0–0.06
35–50	22	0.14	0.08–0.21	14	0.08	0.02–0.15
≥ 50	106	0.76	0.61–0.95	20	0.16	0.06–0.26

Note: Please see the full reference list of the article for more information.

CMR, crude mortality rate; CI, confidence interval.

There was a noticeable change in the reported causes of mortality in 2022 as compared to 2021. Deaths from COVID-19 dropped dramatically from 9.6% to 0% (*p* = 0.02). Malaria and febrile infections increased from 10.7% to 32.3% (*p* < 0.001). Additionally, maternal fatalities increased from 0.6% to 6.2% (*p* < 0.05). In contrast, reported mortality from diabetes, cancer and cardiovascular illnesses decreased from 17.5% to 4.6% (*p* < 0.05). Variable but non-significant alterations were observed in other causes, such as respiratory disorders, accidents, trauma, violence, malnourishment and unidentified causes (Table 5).

**TABLE 5:** Reported causes of death and comparison between 2021 and 2022 surveys.

Cause of death	2021^11^		2022		Total	
	*n*	%	*n*	%	*n*	%
COVID-19	17	9.6	0	0.0	17	7.0
Febrile illness and/or Malaria	19	10.7	21	32.3	40	16.5
Diarrhoea	4	2.3	2	3.1	6	2.5
Other respiratory diseases	12	6.8	2	3.1	14	5.8
Malnutrition	1	0.6	0	0.0	1	0.4
Maternal deaths	1	0.6	4	6.2	5	2.1
Accident, trauma, and/or violence	12	6.8	3	4.6	15	6.2
Cardiovascular diseases, cancer, and/or diabetes	31	17.5	3	4.6	34	14.0
Others	35	19.7	11	16.9	46	19.0
Unknown	45	25.4	19	29.2	64	26.4

Note: Please see the full reference list of the article for more information.

## Discussion

Despite limited change in vaccination coverage and reported cumulative incidence of confirmed cases between the two serosurveys, we found a nearly sixfold rise in SARS-CoV-2 seroprevalence between August 2021 and September 2022. The period between serosurveys was characterised by the occurrence of a major COVID-19 wave marked by the Cameroonian debut of the omicron (B.1.1.529) variant, followed by a minor wave. The emergence of omicron as the dominant variant may explain the increased seroprevalence observed during this period as omicron was highly transmissible despite low pathogenicity as compared to the other variants.^24,25,26^ Other studies reported similar findings, including a 2022 meta-analysis that demonstrated increased seroprevalence after variant emergence. In Africa, specifically, prevalence increased stepwise from 3.5% in June 2020 to 86.7% in December 2021, with each new step correlating with the emergence of a new dominant variant.^27,28^ In January 2022, Cameroon hosted the African Cup of Nations football tournament, a major event that attracted an important number of supporters from 24 African nations and led to large concentrations of people with limited public health and safety measures compliance. This environment could have contributed to a surge in seroprevalence, despite national Cameroon COVID-19 data failing to demonstrate a case surge during that event period.^29^ One limitation of our study is that our testing modalities could not differentiate between antibody responses to infection versus vaccination. The comparable levels of IgM seroprevalence in both surveys suggest a consistent rate of transmission during the respective periods. The discrepancy between the reported cumulative incidence of confirmed cases in 2022 and the seroprevalence indicates that a significant number of symptomatic or asymptomatic cases may have been missed, likely because of insufficient national COVID-19 testing policies and/or limited detection capacity, as acknowledged by the Cameroon Ministry of Health (MoH).^6^ The low prevalence of vaccination in our population further suggests that the observed seroprevalence estimates represent mainly infection-induced antibodies. In addition, neutralising antibodies and SARS-CoV-2-specific memory B cells remain in circulation for over 8–10 months after infection,^30^ such that people exposed to SARS-CoV-2 during the 2021 serosurvey may still have detectable antibodies from infection during the 2022 study, which may have led to a slight overestimate in temporal variation of detected SARS-CoV-2 seroprevalence.

There was no significant change in the vaccination coverage between the two surveys. Our finding differs slightly from the data presented by the Cameroon MoH, which reported a small increase in vaccination rate from 6.1% in August 2021 to 8.7% in September 2022.^29,31^ The slight difference in reported vaccination rates could arise from the study design, as our study focused on estimating seroprevalence and did not account for regional variation in expected vaccination coverage as the MoH data, nor variation across urban versus rural residence. Moreover, the MoH information system captures the proportion of individuals vaccinated as the number of doses given in a target population, which could overestimate the true vaccination coverage, as some individuals receiving repeated vaccine doses documented using different vaccination cards would be counted more than once.

We also found a nearly threefold decrease in CMR between the first and second surveys, with significant reductions in mortality rates attributable to both COVID-19 and non-communicable diseases including diabetes, cardiovascular disease and cancer. From the reported gap in COVID-19 detection, it is highly probable that a significant proportion of deaths thought to be from non-communicable diseases were in fact associated with COVID-19. The decrease in COVID-19 associated mortality over time with a concomitant increase in infection-induced seroprevalence likely reflects naturally acquired host immunity. Previous studies with seasonal coronaviruses have well-demonstrated that infection-induced antibodies correlate with some protection against re-challenge.^32,33,34,35^ In addition, decreased mortality may have been driven by factors indirectly impacted by recovery from the pandemic, such as increased access to healthcare, improved communication about COVID-19, increased health system capacity and full restoration of preventative health services.

Our study had several strengths, including the study design, which ensured a nationwide representative sample, allowing our findings to be generalised. Few other seroprevalence studies in Africa have been conducted with such representative samples. In addition, we used a probabilistic sampling method, which accounted for the geographic variation of seroprevalence. However, the two different testing modalities we used to estimate seroprevalence provided significantly different results, with ELFA estimated seroprevalence 20% higher than RDT estimates. Similar disparities have been observed in other seroprevalence studies,^24,27,36^ and likely result from lower RDT sensitivity in newly-contracted infections and higher detection thresholds than serological immunoassays, like ELFA, resulting in lower seroprevalence estimates when using RDTs compared to ELFA. In addition, we used four different RDT products in this study, which sensitivities ranging from 68.6% to 93.4%, were markedly lower than the 98.9% sensitivity of our ELFA test.^37^

Our study also had several limitations. The multiplicity of tools that we used for seroprevalence detection may have introduced some variability in the obtained results. Such variability can unpredictably increase the margin of error in our estimations. Also, the uncertain portion of the sample that was excluded or declined participation in the seroprevalence survey could have resulted in a selection bias, especially if exclusions differed from participants in their serological status and other key characteristics. Another limitation is that the mortality recall periods were up to one calendar year, which could be a potential source of recall bias if participants were unable to remember the details surrounding their household member’s death. Furthermore, the probable causes of death were reported verbally without being confirmed using official records. Recall bias or social bias around mortality could have resulted in an underestimation of COVID-19 deaths as the contribution of COVID-19 to some deaths might have been missed by household members, deliberately omitted or reported as ‘unknown’ because of COVID-19-related stigma. Similarly, vaccination status was assessed by verbal report without confirmation, which could have resulted in over- or underestimation of vaccination rates from misattribution or recall bias.

Despite these limitations, our study results are critical to understanding the dynamics of SARS-CoV-2 infections in the general population of Cameroon and provide meaningful implications for a better understanding of the disease’s evolution in Africa.

## Conclusion

This study revealed a significant rise in SARS-CoV-2 seroprevalence from 2021 to 2022, despite no substantial change in vaccination coverage. The emergence of the omicron variant and the African Cup of Nations event may have contributed to the increased seroprevalence observed, with seroprevalence estimates mainly reflecting infection-induced antibodies. The study also identified a notable decrease in CMRs between the two surveys, with reductions in mortality rates attributable to both COVID-19 and non-communicable diseases.

Immunity in our population might have been acquired from repeated subclinical infections such that the reoccurring infection has triggered a potent immune response with detectable seroprevalence in most instances, sometimes with clinical manifestation but only rarely leading to death. Our study reinforces the need for improved detection capacities and locally tailored solutions to address global challenges as seen during the COVID-19 pandemic in Cameroon.
